# Action Mechanisms of Curcumin in Alzheimer’s Disease and Its Brain Targeted Delivery

**DOI:** 10.3390/ma14123332

**Published:** 2021-06-16

**Authors:** Duygu Ege

**Affiliations:** Biomedical Engineering, Boğaziçi University, Rasathane Cd, Kandilli Campus, Istanbul 34684, Turkey; duygu.ege@boun.edu.tr; Tel.: +90-530-232-79-12

**Keywords:** AD, curcumin, lipid-based carriers, PLGA, in vivo, drug delivery, antioxidant

## Abstract

AD is a chronic neurodegenerative disease. Many different signaling pathways, such as Wnt/β-catenin, Notch, ROS/JNK, and PI3K/Akt/mTOR are involved in Alzheimer’s disease and crosstalk between themselves. A promising treatment involves the uses of flavonoids, and one of the most promising is curcumin; however, because it has difficulty permeating the blood–brain barrier (BBB), it must be encapsulated by a drug carrier. Some of the most frequently studied are lipid nanocarriers, liposomes, micelles and PLGA. These carriers are further conjugated with brain-targeting agents such as lactoferrin and transferrin. In this review paper, curcumin and its therapeutic effects, which have been examined in vivo, are analyzed and then the delivery systems to the brain are addressed. Overall, the analysis of the literature revealed great potential for curcumin in treating AD and indicated the challenges that require further research.

## 1. Introduction

Alzheimer’s disease (AD) is a chronic neurodegenerative disorder which is characterized by confusion, memory loss, and cognitive decline [1,2,3,4]. By 2050, it is expected to afflict 115 million people [5,6]. The onset is hard to diagnose because degenerative physiological changes in the brain can begin around 30 years before the appearance of symptoms [7,8]. 

The hallmarks of AD are neurofibrillary tangles of tau proteins and Amyloid-β (Aβ) “senile” plaques that build up in the brain [9,10]. Hyperphosphorylation of tau proteins leads to unbound phosphor-tau which causes its aggregation [11]. The β-amyloids are spontaneously aggregating peptide chains of 39–43 amino acids (4 kDa) that have a cross-beta sheet quaterna structure [12,13]. Aβ species include soluble monomers, dimers, oligomers, and insoluble fibrils/aggregates and plaques [14,15]. Aβ is derived from a large protein called amyloid precursor protein (APP). The cleavage of Aβ from APP by β-secretase enzyme instead of α secretase eventually leads to formation of Aβ40 and Aβ42 (β-amyloid 40 and β-amyloid 42) [16,17]. Soluble Aβ peptide is normally secreted from nerve cells, and any excess is cleared. Initially, microglia cells remove Aβ aggregates; however, the clearance eventually fails due to chronic inflammation [18,19]. Aβ self-aggregates form oligomers and amyloid fibrils. Gradually, the β-amyloid subspecies changes from a soluble protein to insoluble fibrils and senile plaques. Many studies have shown that soluble dimeric and oligomeric Aβ species are more neurotoxic than insoluble deposits [14,20]. Aggregates of Aβ also contribute to neuronal damage. A reversible aggregation occurs due to physical hydrophobic stacking, and an irreversible aggregation takes place due to covalent cross-linking [21,22,23]. In the brain, aggregates are found in the hippocampus and subventricular zone (SVZ) and then progress into the neocortex [24,25].

The brain has a high susceptibility to oxidative damage because it accounts for so much of the body’s consumption of oxygen, around 20% [3,26,27]. The oxidation of proteins, lipids, and DNA by increased amounts of reactive oxygen species (ROS) damages neurons and ultimately leads to neuronal death [28,29,30,31]. ROS increases lipid peroxidation, nitrite levels and decreases the activity of antioxidants like glutathione, catalase, superoxide dismutase (SOD) and glutathione-S-transferase, which may lead to AD [30,32,33]. Neurons are protected against oxidative stress by enzymatic antioxidants (catalase, SOD, glutathione reductase and glutaredoxin) and non-enzymatic antioxidants (vitamin C, vitamin E, selenium, zinc, polyphenol, carotenoids, and glutathione) [34,35]. 

Cholinesterase breaks down acetylcholine, so acetylcholine inhibitors may improve symptoms of memory loss [5,36]. Currently, AD treatments include acetylcholine esterase inhibitors (donepezil, tacrine, rivastigmine, and galantamine) and N-methyl-d-aspartate (NMDA) receptor antagonist memantine. However, these drugs only provide symptomatic relief [9,37]. Moreover, sulfonated anions, benzofuran derivatives, and polyphenol-based compounds show promise [25,38]. One polyphenol is curcumin from *Curcuma longa* (the turmeric plant).

There is a tremendous amount of work on the beneficial effects of curcumin against AD [39,40,41]. The relevant literature was compiled using Web of Science, PubMed, Scopus, and Google Scholar without any year limitation. The keywords used were as follows: Alzheimer’s disease, brain, drug delivery, curcumin, turmeric, in vivo and animal studies. In the first part of this review paper, the in vivo studies carried out with curcumin and its action mechanisms are presented. In the second section, carriers used for curcumin and brain targeting agents are covered. Finally, current limitations and future work are discussed. 

## 2. Curcumin

Curcumin (5-hydroxy-1,7-bis(4-hydroxy-3-methoxyphenyl)-1,4,6-heptatrien-3-one) is a plant derived antioxidant with 2 phenol groups [39,40,41] and has been found to be therapeutic for a variety of diseases: neurological, respiratory, cardiovascular, urinary, reproductive, digestive, musculoskeletal, skin, gum, endocrine, and autoimmune (e.g., rheumatic arthritis) [42,43]. It was also found effective for autoimmune disease, such as rheumatic arthritis [44,45,46]. Curcumin’s neurological benefits are applicable to AD, Parkinson’s disease, brain tumors, multiple sclerosis, traumatic brain injuries, ischemia, and depression, which makes it one of the most promising natural therapeutic agents against AD [43,47]. It is one of the most promising natural therapeutic agents against AD [48,49]. In India, the lower prevalence of AD compared to other countries (4.4 times lower than United States) has been attributed to a higher consumption of curcumin [50,51]. The literature suggests that it works via multiple mechanisms against AD [52,53,54]. The literature suggests that it inhibits β-amyloid, phospho-tau, and acetylcholineeasterase creation; modulates microglia; and chelates metal [4,55]. It also has antioxidant activity several times higher than that of vitamin E [51,55].

Curcumin scavenges ROS [30,32,56,57] and increases the levels of SOD, Na^+^-K^+^ ATPase, catalase, glutathione, and mitochondrial complex enzyme. It also decreases lipid peroxidation, usually by reducing malondialdehyde (MDA), nitrite, and acetylcholinesterase levels [28,58]. It also protects mitochondria against peroxynitrite in nigrostriata. This way, it is thought to protect the brain against AD [59,60].

**Figure 1:**
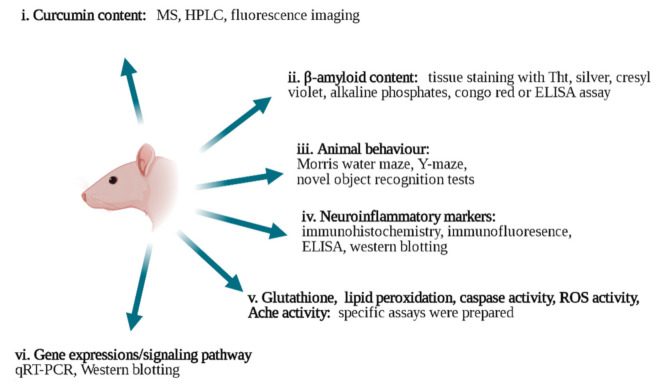
In vivo characterization techniques applied to study the effects of curcumin on AD (drawn in Biorender). **i**. Curcumin content: MS [6], HPLC [58,59,75], and fluorescence imaging [21,41] techniques. **ii**. β-amyloid content: thioflavin T (Tht) staining [61,76,77,78,79] silver staining [39,80], cresyl violet [81], alkaline phosphates [82,83], congo red [78,79], and ELISA assay [2,18,84]. **iii**. Animal behavior tests: Morris water maze [32,39,85], Y-maze [5,86,87], and novel object recognition tests [5,66]. **iv**. Neuroinflammatory markers: immunohistochemistry [87,88,89,90], Western blotting [91], qRT-PCR analysis [91,92] and ELISA assay [86,92]. **v**. Glutathione [3,93], lipid peroxidation [56,87], caspase activity [94,95], ROS activity [96], Ache activity [97,98,99] assays were used. **vi**. Gene expressions and signaling pathways: qRT-PCR [84,100] and Western blotting [39,84,101].

The use of curcumin, however, faces obstacles. First, it has exceptionally low water solubility (11 ng/mL in aqueous buffer pH 5.0), which means that it is eliminated from the body quickly and has a low oral bioavailability. It is soluble in dichloromethane [61,62], ethyl acetate [63,64], ethanol [65], acetone [66,67], chloroform [68], dimethyl sulfoxide [69], and methanol [70]. Due to its low water solubility, it is eliminated from the body at a fast rate, and therefore, its oral bioavailability is quite low. Additionally, it is chemically unstable [71,72,73]. Many strategies have been tried to improve the efficiency of curcumin for AD, and these will be presented in this paper [39,74]. Figure 1 shows the in vivo techniques used to analyze the effectiveness of different strategies. 

### 2.1. Therapeutic Effects of Curcumin

#### 2.1.1. Neurogenesis

Neurogenesis occurs during adulthood when neural stem cells differentiate, especially in the hippocampus [66]. Curcumin has strong neuroprotective effects [74,102]. It regulates neurogenesis in the hippocampus and protects dopaminergic neurons by activating nuclear factor erythroid 2-related factor (Nrf2), the master regulator of the antioxidant response [24,26,34,103] It also upregulates β-tubulin, neuroD1, doublecortin (DCX), neurogenin, neuroligin, and neuregulin and downregulates the signal transducer and activator of transcription 3 (Stat3) [104]. These processes lead to improved memory retention [84,89,105,106,107] as was achieved with the application of 50 mg/kg of curcumin for 3 months or 200 mg/kg of curcumin for 45 days to transgenic mice [106,108]. Yin et al. [109] demonstrated that curcumin improved cognitive function via the promotion of axonal regeneration. 

The Bcl-2 family of proteins is known to play a role in neuronal cell death [16]. Curcumin reduced Bax, caspase-3, cytochrome complex and Poly [ADP-ribose] polymerase 1 (PARP-1), and apoptotic proteins but upregulated the anti-apoptotic protein Bcl-2 [87]. Interestingly, Begum et al. [91] showed that although low brain concentrations of curcumin (500 nM) stimulated neurogenesis, high brain concentrations (10 µM) inhibited neurogenesis and neuroplasticity. Therefore, the choice of concentration of curcumin should be carefully chosen.

Li et al. [110] carried out PCR studies on the The Notch signaling pathway, which is vital for the development of the nervous system, and observed that curcumin used it to promote neural stem cell differentiation in APP/PS1 mice. The other major neuronal communication channel for the formation of neuronal circuits is the Wnt signaling pathway, which is also altered during AD. The pathway promotes cognition and synaptic plasticity [111], and the Wnt/β-catenin is involved in the development of cortical and hippocampal neuroepithelium [24]. Deficiency of Wnt leads to activation of GSK-3β, which may play a role in neuronal death. In a study, the interaction of curcumin with Wnt inhibitor factor (Wif-1), GSK-3β and Dickkopf (Dkk-1) led to the activation of the Wnt/β-catenin pathway which plays a role in synapses stability [16,24]. A treatment with 50 mg/kg of curcumin also ameliorated synaptic transmission via the CaMKII-dependent pathway [66].

#### 2.1.2. Inhibition of Neuroinflammation

Curcumin has a high-affinity for binding to senile plaques [112], and many studies showed that it inhibited Aβ plaque aggregation [9,24,60,113,114]. while decreasing amyloid levels and plaque pathology [51]. Curcumin accomplishes this by breaking down the β-sheet structure of Aβ and the tau aggregates [9]. A high dose of (50 mg/kg/day) of curcumin was found to be effective, but no effect was observed with the application of 10 mg/kg/day [66]. In another study, involving 50 mg/kg/day of curcumin-loaded solid lipid nanocapsules (Cur-LNCs), cognition improved by 90%, acetylcholinesterase inhibition was 52%, and neurological scoring improved by 79% [58]. On the other hand, Cheng et al. [26] showed that even at a low concentration (0.1–1 µM), curcumin can inhibit Aβ fibril formation [26]. 

Neuroinflammation is detrimental to neurons, and prolonged neuroinflammation is a leading characteristic of AD [87]. Normally, microglial cells and astrocytes protect neurons against cytotoxicity [115], and researchers have observed an increase in activated microglia and astrocytes in Aβ-infused rats [51,66,104]. This activation could trigger the (M1 phenotype) processes, which release pro-inflammatory cytokines such as IL-6 and TNF-α [116].

**Figure 2:**
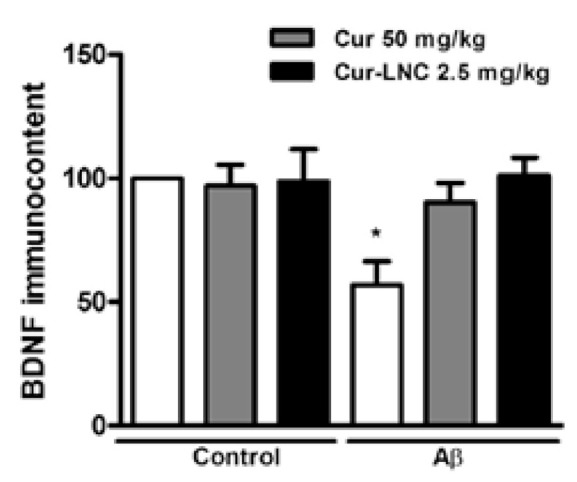
BDNF immunocontent after application of Aβ(1-42) and curcumin. * Significantly different from all other study groups (*p* < 0.05) Significantly different from all other study groups (*p* < 0.05) [66]. Reproduced from [37] with the permission from Elsevier.

In the studies, either transgenic mice were used, or many different agents were applied to induce AD to the rodents. With the administration of Aβ, Hoppe et al. [66] observed triggering of pro-inflammatory processes from increase in glial fibrillary acidic protein (GFAP). A dose of 50 mg/kg/day curcumin or 2.5 mg/kg/day curcumin-loaded lipid-core nanocapsules was followed for 10 days and reduced GFAP immunocontent [51,66]. The same doses of curcumin also diminished the secretion of proinflammatory IL1b cytokines, IL-6 and TNF [66,104]. Hoppe et al. [66] found that brain-derived neurotrophic factor (BDNF) supported neuronal survival. Aβ(1-42) injection led to reduction of BDNF while curcumin and curcumin-loaded lipid-core nanocapsules (Cur-LNC) increased BDNF survival. Figure 2 shows these results. 

#### 2.1.3. Signaling Pathways

Curcumin inhibits multiple dysregulated cell-signaling pathways [48], most of which engage in crosstalk [53,54]. Tau phosphorylation is normally regulated by insulin and insulin-like growth factor (IGF-1), and any impairment may lead to hyperphosphorylation of tau. In the study of Işık et al. [117], Wistar rats were treated with streptozotocin and then given 300 mg/kg of curcumin. The results showed that streptozotocin led to a decrease in IGF-1 levels that were upregulated after treatment with curcumin.

PI3K/Akt/GSK-3β signaling pathway is also directly affected by Aβ exposure [16]. The PI3K/Akt pathway promotes neuroprotection against AD, and Akt phosphorylation can be activated by curcumin [66]. AKT protein kinases phosphorylate and inactivate GSK-3β, which is a serine/threonine protein kinase associated with the progression of AD [118]. Therefore, the administration of curcumin led to deactivation of GSK-3β which in turn reduced Aβ production and build-up of plaques [24,66]. Acetylcholine affects learning, memory, and other cognitive functions. The reduced levels of acetylcholine may cause AD [119]. A link was observed between the activation of GSK-3β and impaired cholinergic function. With the deactivation of (phosphorylation) GSK-3β after the application of curcumin, the cholinergic activities were increased [120].

Nuclear factor kappa B (NF-κB) also increases in the brains of AD patients [121]. Khan et al. [87] administered lipopolysaccharide(LPS) to an animal model to induce neuroinflammation and observed increase of expression of GFAP, p-NF-*κ*B, Iba-1, TNF-*α* and IL-1β. With the administration of curcumin, these cytokines were downregulated via the JNK/NF-κB/Akt pathway [87,122]. Similarly, Wang et al. [123] showed that curcumin inhibited JNK-3 phosphorylation. Karlstetter et al. [104] inhibited LPS-induced cyclooxygenase-2 (COX-2) by administration of curcumin. This occurs by hindering NF-*κ*B, activator protein 1 (AP1), and STATs [124]. Curcumin also hindered matrix metalloproteinase-9, nitric oxide synthase (iNOS), IL-8 and anti-apoptotic proteins that are also regulated by NF-κB [125]. 

NF-κB inhibition was also observed in the study of Giacomeli et al. [85]. After treating albino mice with 50 mg/kg curcumin for 15 days, the downregulation of NF-κB signaling led to GSK-3β-mediated inhibition of Beta-secretase 1 (BACE1), which ultimately reduced Aβ plaques [126]. Zheng et al. [127] showed that the strong inhibition of BACE1 expression reduced Aβ production after the administration of 150 mg/kg of curcumin for 60 days.

Moreover, recent studies showed that mTOR inhibitors show potential for the treatment of neurological disease [128]. The activation of mTOR may increase Aβ production by upregulating β- and γ-secretase cleavage, and Cai et al. [129] indicated that mTOR regulates other relevant signaling pathways, including GSK-3β and IGF-1. Therefore, it was suggested that the mTOR pathway may be responsible for AD. Su et al. [53] showed that 5.24 µg/mL of the curcumin analogue (TML-6) inhibited the phospho-mTOR pathway and decreased Aβ production. Similarly, Wang et al. [130] reduced Aβ levels by downregulating the PI3K/Akt/mTOR signaling pathway.

Teter et al. [18] carried out PCR studies after the oral administration of a low dose of curcumin (160 ppm) and observed an increase of expression of innate immune genes *TREM2, TyroBP* and *Arg1*, while a decrease was observed in CD11b, iNOS, COX-2, C1q, and the sialic acid-binding “siglec” receptor CD33. However, a high dose of curcumin (300 ppm) did not alter *TREM2* levels. This showed that a lower dose of curcumin was found to be more effective for upregulating TREM2.

For late onset AD, APOE4 allele is a major risk factor. Female APOE3- and APOE4-targeted gene replacement mice were fed western diet for 11 months, and then, curcumin was added to their diet for 3 months. For the APOE3 mice, curcumin promoted the expression of transcription factors GABPa, PPARc, and TFAM and PGC1, the master regulator of mitochondrial biogenesis and function. However, APOE4 mice did not express these transcription factors [108]. 

#### 2.1.4. Metal Chelation

Metal ions are required to regulate numerous biological activities, but when APP function is disrupted, metal concentration in the brain rises. Studies indicate that metal ion imbalance leads to neurodegeneration and cell apoptosis [16], but curcumin is a potent chelator of redox-active metal ions [131]. For example, aluminum causes Aβ oligomerization, and aluminum-aggregated Aβ binds to the surface of neurons [132]. Under exposure with aluminum, a high concentration of aluminum is found, especially in the hippocampus [133]. In the mouse brain, aluminum decreased acetyl cholinesterase activity and in rabbits it caused learning deficits in the Morris water maze task. With the application of curcumin, aluminum concentration in the hippocampus was significantly decreased [32]. In another study, aluminum treatment for four weeks induced significant increases in the expressions of BACE1 and APP as well as inflammatory cytokines associated with a reduction in presenilin 1 and 2. With the treatment of 200 mg/kg of curcumin, COX expression decreased in only 24 h [101]. A number of other studies showed that the corpus striatum, cortex, and hippocampus are vulnerable to arsenic toxicity in connection with behavioral and neurochemical abnormalities. Curcumin also reduced the toxic effects of arsenic [134,135], acrylonitrile [136], lead [137], cadmium [138], *1*-methyl-*4*-phenyl-*1,2,3,6*-tetrahydropyridine [139], iron [140], acrylamide [141], and ion irradiation [34].

#### 2.1.5. Co-Delivery of Curcumin with Other Agents

**Figure 3:**
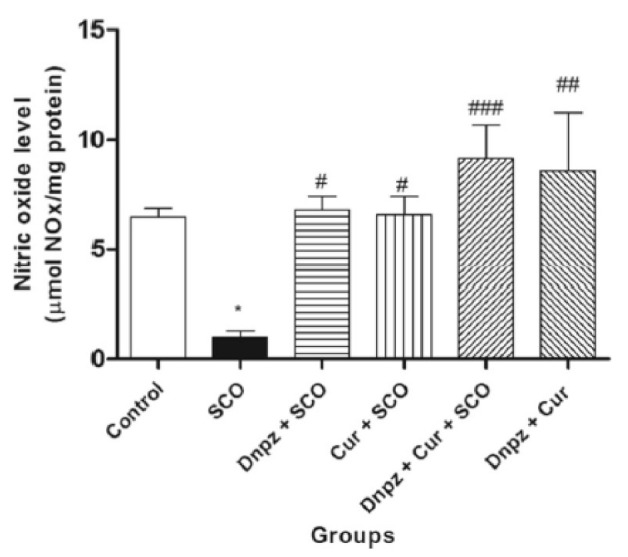
Effect of combination of 50 mg/kg curcumin and 2.5 mg/kg donezepil (Nnzp) after application of scopolamine (SCO) on NO levels. (* *p* < 0.05 vs control; # *p* < 0.05, ## *p* < 0.01, ### *p* < 0.001 vs SCO) [5]. Reproduced from [5] with the permission from Springer.

In many studies, curcumin is administrated with other drugs or antioxidants. In the study of Akinyemi et al. [5] albino Wistar rats underwent induced memory loss by the application of 1 mg/kg scopolamine, and 50 mg/kg curcumin taken orally together with 2.5 mg/kg donezepil (a cholinesterase drug) for 7 days. Curcumin and donezepil had a synergistic effect on memory after novel object recognition and Y-maze tests. The results suggested that this improvement was attributed to the inhibition of cholinesterase which led to improvement of cholinergic neural transmission. The deamination of the neuromodulator adenosine by the ADA enzyme is also associated with memory loss, but the authors also found that a combination of curcumin and donezepil hindered ADA activity. Finally, nitric oxide (NO) was also increased by the combination of curcumin and donezepil, which may have led to enhanced memory function [5]. Figure 3 shows change of NO levels during the study.

Figure 3 shows that scopolamine significantly reduced NO levels and that the combined application of donezepil and curcumin increased NO levels even higher than for the control group [5]. 

In the study of Sharman et al. [12], 83 mg/kg of curcumin was combined with (−)epigallocatechin-3-gallate (EGCG), docosahexaenoic acid (DHA) and α-lipoic acid (ALA) and fed to 6-month-old Tg2576 transgenic mice for 12 months. Interestingly, the study indicated that the combination of curcumin with other antioxidants did not enhance its effect, meaning that a synergy was not present. In another study, curcumin and ferulic acid was orally administered to PPswe/PS1dE9 transgenic mice. Both soluble and insoluble Aβ plaques decreased with co-administration of the antioxidants. Moreover, the pro-inflammatory marker IL-1β decreased further [142]. 

### 2.2. Targeting the Brain

#### 2.2.1. Effect of Concentration and Particle Size 

Schiborr et al. [143] indicated that when they administered 50 mg/kg of curcumin, they did not find any trace in the rodent brain, but because curcumin has low toxicity, the dosage can be increased [108]. Accordingly, Zhang et al. [118], applied 100 mg/kg of curcumin and observed that it was delivered more effectively than 50 mg/kg.

**Figure 4:**
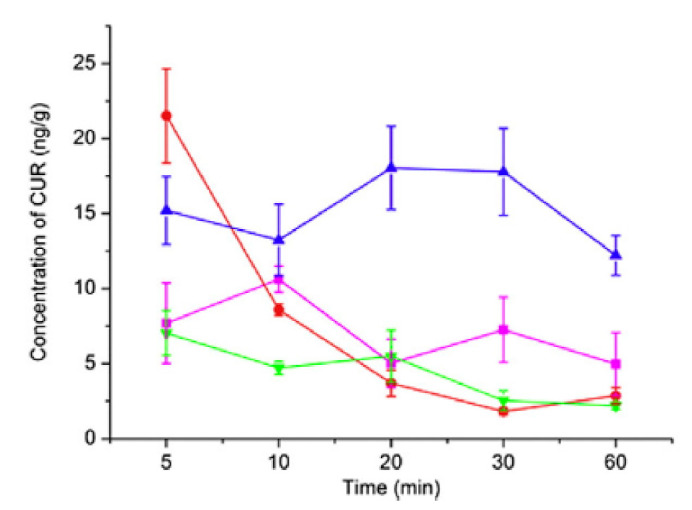
Concentration of different sizes of 2 mg/kg curcumin suspension (CUR-NS) in the brain over time [144] (pink: curcumin solution, red: 20 nm Curcumin-NS, blue: 70 nm CUR-NS and green: 200 nm: CUR-NS). Reproduced from [92] with the permission from Elsevier.

Bi et al. [144] studied the effect of different particle sizes of curcumin in suspensions and found out that the concentration of curcumin was initially the highest for the smallest size. On the other hand, a 70 nm curcumin suspension (CUR-NS) had the highest concentration in the brain for the longest duration. Figure 4 shows the different concentration of curcumin in the brain over time.

#### 2.2.2. Drug Delivery Devices 

**Figure 5:**
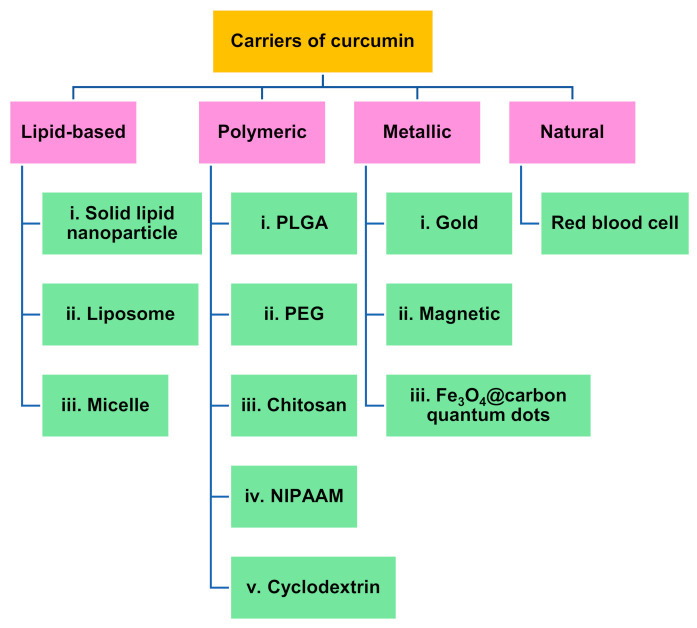
Carriers used for the delivery of curcumin for Alzheimer’s treatment for in vivo studies [6,39,60,75,145,146,147,148].

Only small molecules with low molecular weight and high lipid solubility can pass through the blood–brain barrier; therefore, liposomes, polymeric nanoparticles (polyethylene glycol and poly(lactide-co-glycolide), cyclodextrins, solid dispersions and conjugates) are used to increase the permeability of curcumin [39,40]. Figure 5 shows the carriers used for delivering curcumin to the brain. 

Table 1 shows the in vivo studies with curcumin with targeting agents for AD. According to Table 1, different curcumin loadings were applied for different durations. Moreover, the rodents and drug administration methods were varied. Nanocarriers with a size of less than 10 nm underwent renal filtration. Nanoparticles with a diameter of 50–200 nm remained in the capillaries. Lipid-based particles larger than 150 nm can be removed by phagocytes [149,150] The table shows the wide range of nanoparticle size: co-crystal micelle crystals had the smallest particle size, which might be too small for drug delivery to the brain. In this case, PLGA and other lipid nanoparticles may be more suitable. After tissue extraction, in vivo bioavailability and distribution of curcumin were extensively examined with analytic methods such as MS and HPLC [58,59]. 

The studies showed that curcumin was mainly distributed in the liver, spleen, and lung. In the brain, it was chiefly found in the hippocampus. Nanoparticulation increased the retention time in the cerebral cortex by 96% and in the hippocampus by 83% [59]. When curcumin was combined with nanostructured lipid carriers, its organ distribution increased tremendously as the intestinal epithelium and gastrointestinal tract acted as a barrier to curcumin absorption.

**Figure 6:**
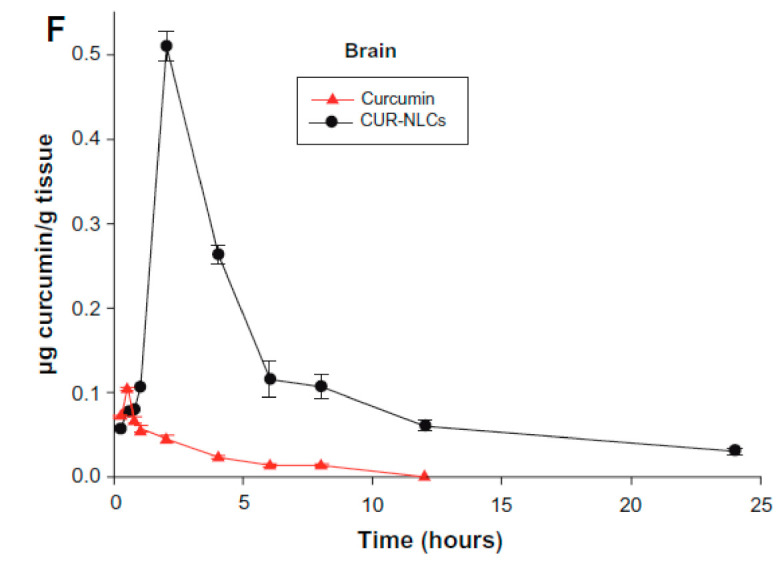
Curcumin nanostructured lipid carrier (Cur-NLC) tissue concentration in the brain with time [75].

When curcumin was combined with nanostructured lipid carriers, curcumin’s organ distribution increased tremendously. The intestinal epithelium and gastrointestinal tract acts as a barrier to curcumin absorption. Encapsulation of curcumin with nanostructured lipid carriers increased the bioadhesion at the wall of the gastrointestinal tract. Curcumin concentration was determined with use of HPLC assay [75]. Figure 6 shows concentration of curcumin in the brain after administration of 80 mg/kg curcumin encapsulated drug. 

With the application of curcumin with a nanostructured lipid carrier, the amount of curcumin in the brain significantly increased, and its retention time in the brain was prolonged. In addition to the higher distribution and greater retention time, solid lipid curcumin also increased anti-inflammatory and neuroprotective effects [81]. 

Maiti et al. [81] showed that, as well as higher brain permeability, solid lipid curcumin particles had a higher affinity for Aβ plaques than curcumin did. Aβ plaques decreased by 50% in 5 days after the application of 50 mg/kg of curcumin nanoparticles. Cresyl-violet staining showed that the plaques were found to be reduced in the prefrontal cortex and hippocampus. Curcumin also maintained neuron viability, which was observed from BDNF, glial-derived neurotropic factor (GDNF) and nerve growth factor (NGF). Finally, inhibition of GFAP was observed in the prefrontal cortex, hippocampus (CA1, CA3, dentate gyrus), subiculum, entorhinal cortex and striatum. This was attributed to activation of peroxisome proliferator-activated receptor-gamma (PPAR-γ) after an injection of curcumin. 

LPS was used to induce neural inflammation, and after the application of gold particle-functionalized curcumin, the LPS’s proinflammatory effects were diminished [145]. In this study, functionalization of curcumin with gold nanoparticles (20 mg/kg) increased the brain uptake of curcumin.

**Figure 7:**
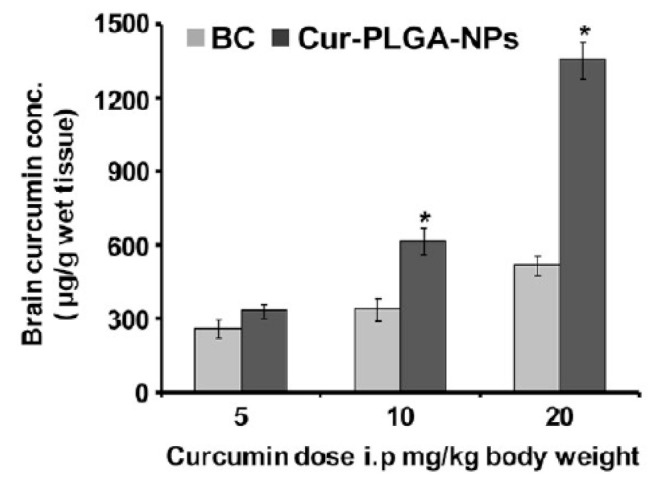
Concentration of drug in the brain for different dosages of uncoated bulk curcumin (BC) and curcumin/PLGA nanoparticles. * *p* < 0.05 versus BC [24]. Reproduced from [24] with permission from American Chemical Society.

Poly(lactide-co-glycolide) (PLGA) has been approved as a promising pharmaceutical delivery platform for its excellent biocompatibility, low cytotoxicity, and biodegradability [162,163]. In some studies, curcumin was loaded in PLGA to treat AD. However, PLGA by itself was not found effective for penetrating the BBB [164]. Figure 7 shows that curcumin concentration in the brain rose significantly after encapsulation in PLGA [24]. Moreover, Ryu et al. [165] showed that when curcumin was co-administered with piperine orally, curcumin levels were increased by 2000% at 45 min after administration.

Thus far, clinical trials have shown insignificant levels of curcuminoids in brain tissue, which indicates the necessity of brain-targeted delivery of efficient concentrations of it [48,166]. Insulin receptors, transferrin and integrin receptors, and receptor-mediated transport are highly abundant on the endothelial cells of the blood–brain barrier. Among these receptors, transferrin is commonly exploited for BBB targeting [154]. Low-density lipoprotein receptors also have overexpression in the brain capillary endothelial cells, which points to the tremendous potential of lipoproteins for brain drug delivery [150]. Thus far, the exploited targeting agents for in vivo studies have been lactoferrin, transferrin, odorranalectin, T807 molecules, Tet-1 peptide, B6 peptide, and N-trimethyl chitosan.

Curcumin-loaded PEG–PLGA with the B6 peptide was produced to target the brain, and encapsulation with PLGA and PEG–PLGA increased its bioavailability by 15.6 and 55.4%, respectively [25]. PLGA internalize into the cells via clathrin-mediated endocytosis [24]. However, the acidic byproducts of degradation may make PLGA encapsulation unsuitable for brain delivery [162,167]. In the study of Jai et al. [154], PEG–PLGA was combined with transferrin (an iron-binding blood plasma glycoprotein) and the peptide Tet-1, the latter of which significantly increased the percentage of the brain’s uptake of 5 mg/kg of curcumin compared to combination with transferrin alone.

In the study of Gao et al. [155], red blood cells were functionalized with T807 and the triphenylphosphine (TPP) cation for intravenous delivery of curcumin to the mitochondria in the brain tissue. T807 is a low-molecular-weight tau positron emission tomography imaging agent, so it can cross the BBB. It also has a strong affinity for phosphorylating tau-positive human brain sections [168]. TPP is one of the most promising mitochondria-targeting agents, as rodents in Morris maze studies demonstrated improved learning and memory when red blood cells were functionalized with T807 and TPP and improved SOD levels. Damage to the hippocampus from ROS activity was reduced as were levels of hydrogen peroxide (H_2_O_2_), γ-glutamyl transpeptidase (γ-GT), and MDA. Moreover, GFAP and Iba-1 decreased, which indicated a decrease in inflammation after drug treatment. [155]. 

Another method used to deliver curcumin is through the nose [169]. It enables curcumin to bypass the BBB to enter the brain and cerebrospinal fluid through the nasal mucosa [147]. Curcumin in the nasal cavity reaches the nasal mucosa and arrives at the olfactory region and trigeminal nerve pathways [6]. In the study of Li et al. [147], PEG–PLGA was conjugated with odorranalectin to improve delivery. Odorranalectin extended the residence time of curcumin in the nasal mucosa and promoted its nasal uptake and increased the bioavailability of curcumin in vivo.

In another study of Zhang et al. [6], PLGA was coated with chitosan to open the tight junctions between nasal mucosal epithelial cells to enable transport to the brain. Curcumin levels in the brain were studied using the LC−MS/MS method, which indicated significantly elevation after coating with chitosan.

Overall, results indicated that some drawbacks exist for the frequently studied carrier systems. PLGA has the disadvantage of fast clearance from the reticuloendothelial system before reaching the brain. Therefore, it is usually conjugated with PEG to increase retention time and enhance curcumin delivery to the brain [170,171]. As discussed earlier, the size of the nanocarriers is critical. Micelles smaller than 50 nm may be excreted before reaching the brain, whereas liposomes may be too large and be removed from the system by phygocytes [160,161]. Among lipid-based nanocarriers, nanoparticles have been shown to be promising; therefore, exploitation of PEG–PLGA or solid lipid nanoparticles after conjugation with targeting agents are encouraging.

In the literature, the applied range of curcumin varied from 0.5 to 300 mg/kg, so it would be beneficial to carry out a systematic study to determine the most effective dosage. The studies also indicated that although the low brain concentration of curcumin stimulated neurogenesis, extremely high brain concentrations inhibited neurogenesis and neuroplasticity. Therefore, the concentration of curcumin should be carefully chosen to optimize its effects on AD.

Thus far, only in vivo studies on the effects of curcumin have been carried out, and these have limitations. For instance, mice may undergo stress during experiments which might result in biased results. Moreover, the translation of data from in vivo studies needs extensive research before any clinical use.

In future studies, curcumin may be combined with other antioxidants to increase its therapeutic effect on AD. Thus far, examinations of signaling pathways are very limited when carriers are used. It would be beneficial to study them to see if curcumin is still viable after delivery. Graphene oxide is another promising drug carrier to enhance delivery to the brain [166,172,173]. Overall, curcumin shows promise for treating AD, but extensive research is needed to understand how to exploit it for clinical use.

## 3. Conclusions

Curcumin was investigated heavily as a treatment for AD. It stimulated neurogenesis via the Notch and Wnt/β-catenin pathways, diminished the secretion of proinflammatory cytokines, and led to the deactivation of GSK-3β, which in turn reduced Aβ production and the buildup of plaques by downregulating the ROS/JNK pathway. Furthermore, downregulation of NF-κB signaling led to GSK3β-mediated inhibition of *BACE1*, which ultimately reduced *A*β plaques. However, the exact mechanism by which curcumin regulated these processes is still unknown.

To increase curcumin’s permeability across the blood–brain barrier, it was encapsulated and conjugated with different agents. The studies indicated that lipid-based carriers and PLGA increased its organ distribution tremendously. The functionalization of curcumin with metallic nanoparticles also enhanced the uptake of curcumin in the brain. Furthermore, the conjugation of carriers with targeting agents, such as Tet-1 peptide, transferrin, lactoferrin, and chitosan increased the blood–brain barrier permeability of curcumin. However, research on the mechanism of curcumin with a delivery vehicle is limited. It would be beneficial to study the important signaling pathways after curcumin is encapsulated with nanocarriers to see if the action mechanism of curcumin is sustained. Overall, the studies indicated that curcumin is a very promising antioxidant for the treatment of AD, and the use of carriers and targeting agents is very effective for enhancing delivery to the brain. 

## Figures and Tables

**Table 1:** In vivo studies involving a carrier and targeting agent for the delivery of curcumin for the treatment of AD.

Carrier	Amount of Curcumin Nanoparticles	Particle Size (nm)	Carrier and Conjugation Details	Animals Used	Main Results
**Metal-based**	200 mg/kg (for 30 days)	N/A	Magnesium oxide nanoparticles	Albino rats (oral delivery)	Declined AChE levels [99]
	20 mg/kg (unknown duration)	N/A	Gold nanoparticles functionalized puerarin	Sprague Dawley lipopolysaccharide (LPS)-induced inflammation (intravenous injection)	Anti-inflammatory effects [145]
**Lipid-based**	2.5 mg/kg (for 10 days)	200	Lipid-core nanocapsules	(male adult Wistar rats) Aβ(1-42)-infused (intraperitoneal administration)	Improved animal memory Anti-inflammatory effects [66]
	4 mg/kg (for 7 days)	N/A	Solid Lipid Nanoparticles	Sprague−Dawley rats (parenteral administration)	Improved animal memory [151]
	10 mg/kg (for 7 days)	28.8	Co-crystal Micelles	Female Sprague-Dawley rats (intranasal delivery)	4.5- and 6-fold enhancement in relative bioavailability of curcumin [60]
	10 mg/kg (applied for 3 weeks)	90.5	Nanostructured lipid carrier and Polysorbate 80 coating (targeting: lactoferrin)	Sprague-Dawley (SD) rats (intravenous injection)	Improved bioavailability of curcumin Aβ plaques decreased [152]
	20 mg/kg (applied for 3 weeks)	75–163	Nanostructured lipid carrier (NLC) (targeting: lactoferrin)	Sprague-Dawley (SD) rats (injection via caudal veins)	Aβ plaques decreased [153]
	25 mg/kg (applied once)	139–514	Solid Lipid Nanoparticles (targeting: N-trimethyl Chitosan)	male Balb/c mice (oral administration)	23% higher bioavailability than curcumin [154]
	50 mg/kg (applied once)	N/A	Solid lipid nanoparticles	Wistar rats (intravenous injection)	Better bioavailability [58]
	10 mg/kg (for 15 days)	N/A	Lipid core nanocapsules	Female Swiss Albino Mice (intracerebroventricular injection)	Anti-inflammatory effects [85]
	80 mg/kg (applied once)	129	Nanostructured lipid carriers	Sprague-Dawley rats (oral administration)	Better bioavailability [75]
	83 mg/kg (every other day for 2 months)	N/A	Solid lipid nanoparticles	5xFAD mouse (intraperitoneally)	Improved animal memory (escape latency: 10 s) [40]
	N/A	207	Nanoliposomes	APPxPS1 mice stereotactic (intracerebral injection)	Aβ plaques decreased [155]
**PLGA-based**	0.5–20 mg/kg (for 5 days	200	PLGA	Wistar rats Amyloid induced (stereotaxic injection)	Aβ plaques decreased [24]
	2 mg/kg (every 2 days for 3 weeks)	128	PLGA-cyclic CRTIGPSVC peptide	(APP/PS1dE9) mice (intraperitoneal injection)	Reduced inflammation, improved memory [86]
	15 mg/kg (for 14 days)	63.2	PEG-PLGA (targeting: transferrin and Tet-1 peptide)	BALB/c mice (intravenous injection)	Improved bioavailability Improved animal memory (Escape latency: 30 s) [156]
	1 mg/kg (applied once)	97	PEG–PLGA/PEG–PBLG Nanoparticles (targeting: odorranalectin)	Male Sprague Dawley (SD) rats (intranasal)	Improved bioavability [147]
	2 mg/kg (applied once)	247	Chitosan-Coated Poly(lactic-co-glycolic acid) Nanoparticles and Hydroxypropyl-β-Cyclodextrin Inclusion Complexes	Male C57BL/6 mice (intranasal)	Better bioavailability [6]
	5 mg/kg (for 10 days)	<120	Red blood cell (RBC) membrane-coated PLGA (targeting: T807 molecules)	Male ICR mice (tail vein injection)	Better bioavailability Improved animal memory (Escape latency: 45 s) Plaques decreased Anti-inflammatory effect [157]
	5 mg/kg (for 10 days)	170	Red blood cell (RBC) membrane-coated PLGA particles (targeting: T807 molecules)	Female ICR mice (tail vein injection)	Improved animal memory Higher bioavailability plaques decreased [158]
	25 mg/kg (for once)	163	PLGA	Male Sprague-Dawley rats (intravenously injection)	Improved organ distribution [59]
	25 mg/kg (for 3 months)	<100	PEG-PLGA (targeting: B6 peptide)	Male APP/PS1 mouse (intraperitoneal injection)	Improved animal memory (Escape latency: 30 s) Higher bioavailability plaques decreased [25]
**PEG-based**	10 mg/kg (for once)	184	D-α-tocopheryl PEG 1000 succinate and Tween 80	Male Wistar rats (intraperitoneal injection)	Improved organ distribution [159]
	13.6 mg/kg (applied once)	213	PEG 400 based gelling system	Male Wistar rats (Intranasal or intravenously)	Bioavailability of curcumin increased with the carrier [160]
**Other polymers**	5 mg/kg (applied once)	152	Poly(n-butylcyanoacrylate) nanoparticle	Male Kunming mice (intravenous injection)	Enhanced transport to the brain [161]
	25 mg/kg (4 weeks)	N/A	N-isopropylacrylamide (NIPAAM), vinylpyrrolidone (VP), and acrylic acid (AA)	Athymic mice (intraperitoneal route)	Higher bioavailability of curcumin anti-oxidant activity increased [68]

## Abbreviations

ALA	α-lipoic acid
AP1	Activator protein 1
AD	Alzheimer’s disease
APP	Amyloid precursor protein
BACE	Beta-secretase 1
BBB	Blood–brain barrier
BDNF	Brain-derived neurotrophic factor
COX-2	Cyclooxygenase-2
Dkk-1	Dickkopf
DHA	Docosahexaenoic acid
DCX	Doublecortin
EGCG	Epigallocatechin-3-gallate
GDNF	Glial derived neurotropic factor
GFAP	Glial fibrillary acidic protein
HE	Hematoxylin and eosin
HPLC	High-performance liquid chromatography
LPS	Lipopolysaccharide
IGF-1	Insulin-like growth factor
MDA	Malondialdehyde
MS	Mass spectrometry
NGF	Nerve growth factor
NO	Nitric oxide
iNOS	Nitric oxide synthase
NMDA	N-methyl-d-aspartate
NF-κB	Nuclear factor kappa B
Nrf2	Nuclear factor erythroid 2-related factor
PPAR-γ	Peroxisome proliferator-activated receptor-gamma
PARP-1	Poly [ADP-ribose] polymerase 1
PLGA	Poly(lactide-co-glycolide)
ROS	Reactive oxygen species
SOD	Superoxide dismutase
Stat3	Signal transducer and activator of transcription 3
Tht	Thioflavin T
TPP	Triphenylphosphine cation
Wif-1	Wnt inhibitor factor
γ-GT	γ-glutamyl transpeptidase
